# Diagnosis of Prostate Cancer with a Neurotensin–Bombesin Radioligand Combination—First Preclinical Results

**DOI:** 10.3390/pharmaceutics16091223

**Published:** 2024-09-19

**Authors:** Maria Bibika, Panagiotis Kanellopoulos, Maritina Rouchota, George Loudos, Berthold A. Nock, Eric P. Krenning, Theodosia Maina

**Affiliations:** 1 Molecular Radiopharmacy, INRaSTES, NCSR “Demokritos”, 15341 Athens, Greece; mp.maria91@hotmail.com (M.B.); kanelospan@gmail.com (P.K.); nock_berthold.a@hotmail.com (B.A.N.); 2 BIOEMTECH, Lefkippos Attica Technology Park NCSR “Demokritos”, 15310 Athens, Greece; mrouchota@bioemtech.com (M.R.); george@bioemtech.com (G.L.); 3 Cyclotron Rotterdam BV, Erasmus MC, 3015 CE Rotterdam, The Netherlands; erickrenning@gmail.com

**Keywords:** radiotheranostics, cancer targeting, neurotensin subtype 1 receptor, gastrin-releasing peptide receptor, Tc-99m radiotracer

## Abstract

**Background:** The concept of radiotheranostics relies on the overexpression of a biomolecular target on malignant cells to direct diagnostic/therapeutic radionuclide-carriers specifically to cancer lesions. The concomitant expression of more than one target in pathological lesions may be elegantly exploited to improve diagnostic sensitivity and therapeutic efficacy. Toward this goal, we explored a first example of a combined application of [^99m^Tc]Tc-DT11 (DT11, N_4_-Lys(MPBA-PEG4)-Arg-Arg-Pro-Tyr-Ile-Leu-OH; NTS_1_R-specific) and [^99m^Tc]Tc-DB7(DB7, N_4_-PEG2-DPhe-Gln-Trp-Ala-Val-Gly-His-Leu-NHEt; GRPR-specific) in prostate cancer models. **Methods:** Accordingly, the behavior of [^99m^Tc]Tc-DT11 was compared with that of the [^99m^Tc]Tc-DT11+[^99m^Tc]Tc-DB7 mixture in prostate adenocarcinoma PC-3 cells and xenografts in mice. The impact of stabilizing both radiotracers by Entresto^®^, as a source of the potent neprilysin inhibitor sacubitrilat, was also investigated. **Results:** The PC-3 cell binding of the [^99m^Tc]Tc-DT11+[^99m^Tc]Tc-DB7 mixture surpassed that of [^99m^Tc]Tc-DT11. Likewise, the PC-3 tumor uptake of the [^99m^Tc]Tc-DT11+[^99m^Tc]Tc-DB7 mixture at 4 h post-injection was superior (7.70 ± 0.89%IA/g) compared with [^99m^Tc]Tc-DT11 (4.23 ± 0.58%IA/g; *p* < 0.0001). Treatment with Entresto^®^ led to further enhancement of the tumor uptake (to 11.57 ± 1.92%IA/g; *p* < 0.0001). **Conclusions:** In conclusion, this first preclinical study on prostate cancer models revealed clear advantages of dual NTS_1_R/GRPR targeting, justifying further assessment of this promising concept in other cancer models.

## 1. Introduction

The overexpression of key biomolecules, such as G-protein coupled receptors (GPCRs), on cancer cells has offered the opportunity to direct diagnostic or therapeutic radionuclides to pathological lesions using suitably designed carriers, such as peptide ligands, with a high specificity [1]. The new field of radiotheranostics has been initiated and established through the advent of radiolabeled somatostatin analogs in the management of neuroendocrine tumors [2,3,4,5]. Currently, this field has been rapidly expanding toward more clinical indications involving other GPCR-targets, such as the management of frequently occurring prostate or breast cancers [6,7,8,9]. Furthermore, the concomitant expression of more than one biomolecular target on cancer cells has been documented by several studies, providing the molecular basis for a multi-targeting approach [10,11]. Thus, the expression of various peptide receptors has been demonstrated in different numbers and combinations in neuroendocrine tumors [10,12], as well as in breast [13,14] or prostate cancer [15,16,17]. Hence, multi-targeting methods have been proposed via the administration of either radioligand cocktails or suitably designed multi-specific agents to patients [18,19,20,21,22,23,24]. Yet, this strategy, no matter how promising, needs to convincingly address a number of challenges before successfully reaching the clinic. For example, sub-optimal pharmacokinetics may occur in the case of high-molecular-weight multimers or due to undesirable accumulation in an increased number of non-disease-related sites in the body physiologically expressing the target(s). When cocktails of biodegradable peptide radioligands are used, the in vivo stability also needs to be considered [25].

We have long been engaged in the development of peptide radioligands for cancer theranostics, with a great part of our activities being focused on gastrin-releasing peptide receptor (GRPR)-directed analogs for diagnosis/treatment of human prostate cancer [7,9,26]. The overexpression of neurotensin subtype 1 receptor (NTS_1_R) in prostate cancer has also been reported in a number of studies, providing the opportunity for double GRPR/NTS_1_R-targeting [27,28,29,30,31,32]. Interestingly, the density and incidence of NTS_1_R expression was found to increase in advanced and androgen-independent stages of prostate cancer [30,32,33], whereas GRPR-expression is more prominent in the early stages of the disease [7,16,34,35]. These findings imply that dual GRPR/NTS_1_R-targeting of prostate cancer may increase the diagnostic sensitivity of pathological lesions using suitable cocktails of bombesin (BBN, Pyr-Gln-Arg-Leu-Gly-Asn-Gln-Trp-Ala-Val-Gly-His-Leu-Met-NH_2_)- and neurotensin (NT, pyr-Glu-Leu-Tyr-Glu-Asn-Lys-Pro-Arg-Arg-Pro-Tyr-Ile-Leu-OH)-based analogs. We decided to explore this possibility using a BBN together with an NT analog, both carrying an acyclic tetraamine (N_4_, 6-(carboxy)-1,4,8,11-tetraazaundecane) at the N-terminus for facile labeling with the prominent single photon emission computed tomography (SPECT) radionuclide Tc-99m. Selection of the two partner radioligands was based on several factors, such as high receptor affinity, good tumor targeting in mice models, low background activity and metabolic stability.

**Figure 1:**
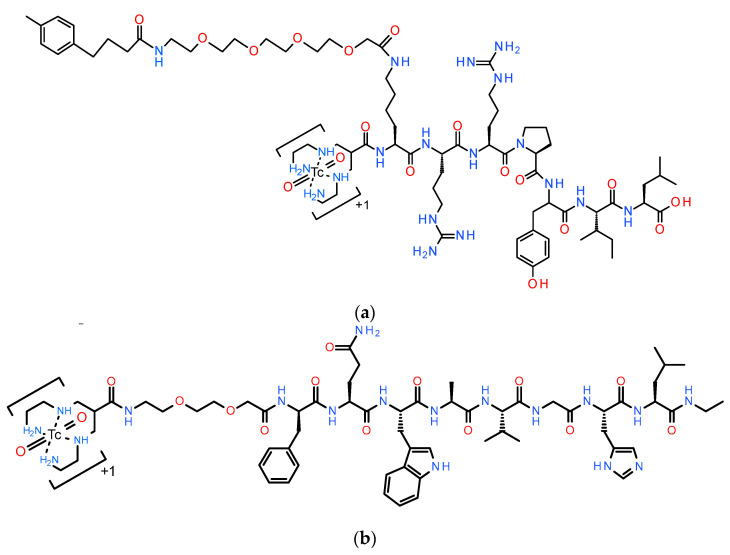
Chemical structures of (**a**) [^99m^Tc]Tc-DT11 (DT11, N_4_-Lys(MPBA-PEG4)-Arg-Arg-Pro-Tyr-Ile-Leu-OH; N_4_, 6-(carboxy)-1,4,8,11-tetraazaundecane; MPBA = 4-(4-methylphenyl)butyric acid; PEG4: 14-amino-3,6,9,12-tetraoxatetradecan-1-oic acid) and (**b**) [^99m^Tc]Tc-DB7 (DB7, N_4_-PEG2-DPhe-Gln-Trp-Ala-Val-Gly-His-Leu-NHEt; PEG2, 8-Amino-3,6-dioxaoctanoic acid).

Amongst several analogs developed and preclinically screened in our studies, [^99m^Tc]Tc-DB7 (DB7, N_4_-PEG2-DPhe-Gln-Trp-Ala-Val-Gly-His-Leu-NHEt; PEG2, 8-amino-3,6-dioxaoctanoic acid; Figure 1a) displayed GRPR-specific uptake in human prostate adenocarcinoma PC-3 xenografts in mice (4.49 ± 1.20%IA/g at 4 h post-injection, pi) vs. a very low background, even from the GRPR-rich pancreas (0.56 ± 0.20%IA/g at 4 h pi), featuring qualities that favor high-contrast imaging with SPECT [36]. Furthermore, [^99m^Tc]Tc-DB7 displayed a fair stability in peripheral mice blood, which could be further enhanced by co-injection of the neprilysin (NEP) inhibitor phosphoramidon (PA) [37,38,39,40]. As a result, significant improvement of radioactivity uptake was achieved in the PC-3 xenografts (to 6.10 ± 1.10%IA/g at 4 h pi; *p* < 0.0001) with minimal increases in physiological tissues [36]. On the other hand, in a number of N_4_-modified NT-analogs labeled with Tc-99m in our studies, we selected [^99m^Tc]Tc-DT11 (DT11, N_4_-Lys(MPBA-PEG4)-Arg-Arg-Pro-Tyr-Ile-Leu-OH; MPBA = 4-(4-methylphenyl)butyric acid; PEG4: 14-amino-3,6,9,12-tetraoxatetradecan-1-oic acid; Figure 1b) as the second partner of our cocktail. This choice was driven by metabolic stability considerations. Two main proteases, NEP and angiotensin-converting enzyme (ACE) [41,42], were previously reported to rapidly degrade NT and its analogs in the biological milieu [43,44,45]. We recently demonstrated that dual administration of NEP and ACE inhibitors stabilized a series of NT-based radioligands, leading to increased uptake in pancreatic tumor models in mice [46,47]. Furthermore, we were able to design side-chain modified NT-analogs showing increased metabolic stability [47]. [^99m^Tc]Tc-DT11 in particular displayed the highest in vivo stability and achieved the highest uptake in NTS_1_R-positive xenografts in mice. Tumor uptake could be further enhanced in the animals treated with Entresto^®^ as a source of the potent NEP inhibitor sacubitrilat (4.48 ± 0.37%IA/g to 6.14 ± 0.08%IA/g at 4 h pi; *p* < 0.0001) [47,48,49,50,51,52]. These results allow us to use just a single, highly specific inhibitor of NEP to in situ stabilize the [^99m^Tc]Tc-DT11+[^99m^Tc]Tc-DB7 mixture, enhancing prospects for clinical translations in future.

In the present work, we decided to first investigate the behavior of [^99m^Tc]Tc-DT11 in PC-3 cells and tumors thereof in mice without or during NEP inhibition. We next assessed the impact of using an equimolar [^99m^Tc]Tc-DT11+[^99m^Tc]Tc-DB7 mixture in the same model, with additional comparisons vs. partial (NTS_1_R or GRPR) or double (NTS_1_R and GRPR) target-blockades. The impact of single NEP inhibition with the aid of the highly specific and potent NEP inhibitor sacubitrilat, released from the registered antihypertensive drug Entresto^®^, was also evaluated (as further detailed in “Metabolic Studies of [^99m^Tc]Tc-DB7 and [^99m^Tc]Tc-DT11 in Mice: Comments–Results” in the Supplementary Materials).

## 2. Materials and Methods

### 2.1. Chemicals and Radioligands

#### 2.1.1. Peptides and Protease Inhibitors

Chemicals were reagent grade; high performance liquid chromatography (HPLC) solvents were HPLC grade. Both NT and [Tyr^4^]BBN were obtained from Bachem AG (Bubendorf, Switzerland). Entresto^®^ pills were purchased from a local pharmacy and were manufactured by Novartis AG, Basel, Switzerland (a total of 200 mg per pill corresponded to 24 mg/26 mg sacubitril/valsartan). Individual doses of 12 mg/200 μL per animal were prepared by grinding the pills to a fine powder and equally distributing them in LoBind Eppendorf tubes (Eppendorf, Wesseling-Berzdorf, Germany); just before use, tap water was added to form a slurry for oral gavage to mice [47]. The peptide conjugates DT11 [47] and DB7 [36] (Figure 1) were synthesized by PiChem Forschungs- und Entwicklungs GmbH (Raaba-Grambach, Austria).

A commercial [^99^Mo]Mo/[^99m^Tc]Tc generator (Ultra-Technekow V4 Generator, Curium Pharma, Petten, The Netherlands) was used as a source of Tc-99m; the latter was retrieved in the form [^99m^Tc]NaTcO_4_ in normal saline by elution of the generator.

#### 2.1.2. Radiolabeling–Quality Control

Both the lyophilized DT11 and DB7 were dissolved to a 2 mg/mL concentration in doubly distilled H_2_O, then distributed in equal 50 μL-aliquots in Eppendorf Protein LoBind tubes and stored at −20 °C. For labeling with Tc-99m, to a LoBind Eppendorf tube containing phosphate buffer (0.5 M, pH 11.5, 50 μL) [^99m^Tc]NaTcO_4_ (420 μL generator eluate) was added. A sodium citrate solution was then added (0.1 M, 5 μL), followed by the respective peptide stock solution (15 μL, 15 nmol) and, eventually, a freshly prepared SnCl_2_ solution in EtOH (10 μL, 10 μg). After a 30 min incubation at room temperature, the pH of the reaction mixture was adjusted to 7.4 by 0.1 M HCl [36,47].

For the quality control, HPLC and instant thin-layer chromatography (iTLC) methods were applied. The HPLC system was based on a Waters Chromatograph linked to binary detection modes, a 2998-photodiode array UV detector (Waters, Vienna, Austria) and a Gabi gamma detector (Raytest RSM Analytische Instrumente GmbH, Straubenhardt, Germany); the system was controlled by the Empower 2 Software (Waters, Milford, MA, USA). For analyses, an XBridge RP-18 (5 μm, 4.6 mm × 20 mm) cartridge column (Waters, Eschborn, Germany) was eluted at a flow rate of 1 mL/min with a linear gradient (system 1): from 100% A/0% B to 40% A/60% B in 20 min (A: 0.1% aqueous TFA and B: MeCN). For iTLC, Whatman 3 mm chromatography paper strips (GE Healthcare, Chicago, IL, USA) were run up to 10 cm from the place of origin with two different systems: (i) 5 M NH_4_AcO/MeOH 1:1 (*v*/*v*) for the detection of reduced hydrolyzed technetium [^99m^Tc]Tc^IV^O_2_ × nH_2_O (R_f_ = 0) and (ii) acetone for the detection of free unreduced [^99m^Tc]TcO_4_^−^ (R_f_ = 10). Sample radioactivity was measured in a γ-counter (automated multi-sample well-type instrument with a NaI(Tl) 3″ crystal, Canberra Packard Cobra^TM^ Quantum U5003/1, Auto-Gamma^®^ counting system; Little Rock, AR, USA). Radioligands in normal saline/EtOH *v*/*v* 9/1 (vehicle) were used in biological experiments; samples thereof were analyzed prior to and following the completion of each experiment.

Procedures with radioactive materials were conducted by trained and authorized personnel only behind suitable lead shielding in dedicated laboratories according to European radiation safety guidelines. Supervision was undertaken by the Greek Atomic Energy Commission (license # A/435/17092/2019).

### 2.2. Cell Studies

#### 2.2.1. Cell Culture

Studies were conducted in human prostate adenocarcinoma PC-3 cells, which are reported to endogenously express the GRPR [53] and the NTS_1_R [21,27]. The cells were obtained from LGC Standards GmbH (Wesel, Germany) and were grown in 75 cm^2^ culture flasks with a vent cap in controlled humidified air containing 5% CO_2_ at 37 °C in a Cell Incubator (Smart Cell-Heal Force, Hong Kong, China). Cells were cultured in Roswell Park Memorial Institute-1640 (RPMI-1640) medium with GlutaMAX-I, which was supplemented with 10% (*v*/*v*) fetal bovine serum (FBS), 100 U/mL penicillin, and 100 µg/mL streptomycin. Passages were carried out at 75–85% confluency using a trypsin/EDTA (0.05%/0.02% *w*/*v*) solution once or twice per week. Culture media were purchased from Selidis, A., Bros–Antisel S.A. (Athens, Greece), while supplements/plastic parts were provided by Bioline Scientific Douros Bros-E. Demagos O.E. (Athens, Greece).

#### 2.2.2. Uptake/Internalization of [^99m^Tc]Tc-DT11 in PC-3 Cells

The day preceding the experiment, PC-3 cells were transferred in 6-well plates (1 × 10^6^ cells in 3 mL culture medium per well), and the plates were positioned in the incubator. On the day of the experiment, the plates were placed on ice and the supernatant was aspirated and discarded. The cells were rinsed with 2 × 2 mL ice-cold internalization medium (IM, RPMI-1640 with 1% FBS) and the plates were placed on the bench. Warm IM (1.2 mL at 37 °C) was added to the wells, followed by either warm IM (150 µL—3 upper wells, total series) or excess NT in IM (150 µL to a final concentration of 1 µM—3 lower wells, non-specific series). In a last step, [^99m^Tc]Tc-DT11 in phosphate-buffered saline with 0.5% bovine serum albumin (PBS-BSA) was added to all plates (250 fmol peptide, ≈100,000 cpm). Cells were incubated for 1 h at 37 °C in an Incubator-Orbital Shaker unit (MPM Instr. SrI, Bernareggio, Milan, Italy). Incubation was interrupted by placing the plates on ice and collecting the supernatants in separate plastic tubes. The cells were rinsed with ice-cold PBS-BSA (1 mL) and the liquid was collected and combined with the respective supernatant tubes. The plates were placed on the bench and incubated 2 × 5 min with acid buffer (2 × 600 µL, 50 mM Gly in 0.1 M NaCl, pH 2.8). Supernatants were again collected and combined in individual tubes (membrane-bound fractions). Cells were rinsed with ice-cold PBS-BSA (1 mL), which was aspirated and discarded. Finally, the cells were treated with 2 × 600 µL 1 M NaOH, detached, collected and combined in separate tubes (internalized fractions). The radioactivity of all tubes was measured in the gamma counter. The percentage of specific membrane-bound and internalized fractions (their sum corresponding to specific cell-uptake) vs. total radioactivity added per well were calculated by subtracting non-specific values from totals. Results were expressed as average values ± standard deviation (sd) from 3 independent experiments performed in triplicate.

#### 2.2.3. Uptake/Internalization of [^99m^Tc]Tc-DT11+[^99m^Tc]Tc-DB7 in PC-3 Cells

In this experiment, two 6-well plates were used per assay instead of one, and the same procedure was followed with a few modifications. Thus, after the addition of warm IM (1.2 mL at 37 °C) to all the wells, the triplicate series was divided as follows: (i) plate 1—upper wells: addition of warm IM (150 µL, total series); (ii) plate 1—lower wells: addition of excess NT in IM (150 µL to a final concentration of 1 µM, NTS_1_R-block); (iii) plate 2—upper wells: addition of excess [Tyr^4^]BBN in IM (150 µL to a final concentration of 1 µM, GRPR-block; and (iv) plate 2—lower wells: addition of excess NT and [Tyr^4^]BBN in IM (150 µL to a final individual concentration of 1 µM, double NTS_1_R/GRPR-block). In a last step, an equimolar [^99m^Tc]Tc-DT11 and [^99m^Tc]Tc-DB7 mixture was added to all plates in PBS-BSA (250 fmol of each analog, ≈100,000 cpm). The same protocol described above was followed, and specific results for the [^99m^Tc]Tc-DT11+[^99m^Tc]Tc-DB7 mixture and the partially blocked groups (either [Tyr^4^]BBN or NT) were calculated after subtraction of non-specific values during the parallel GRPR and NTS_1_R-blockade from the respective total/partially blocked values. Results were finally expressed as average values ± sd from 3 independent experiments performed in triplicate. Statistical analysis was performed with PRISM^TM^ GraphPad–10.2 Software (San Diego, CA, USA) using a 2-way ANOVA with a Tukey’s post hoc analysis, and differences with *p* < 0.05 were considered as statistically significant.

### 2.3. Animal Studies

#### 2.3.1. Metabolic Stability in Mice

The metabolic stability of [^99m^Tc]Tc-DB7 without or during NEP inhibition was studied in two groups of three healthy male Swiss albino mice (30 ± 5 g, NCSR “Demokritos” Animal House, Athens, Greece). Both groups intravenously (iv) received a bolus of [^99m^Tc]Tc-DB7 (100 µL, 55.5–111 MBq, 3 nmol of total peptide in vehicle) via the tail vein; the second group of mice received, by oral gavage, a slurry of Entresto^®^ (12 mg/200 μL per animal) 30 min in advance of radioligand injection [47] (individual doses were determined as detailed in Supplementary Materials). The animals were euthanized 5 min post-injection (pi); blood (0.5–1 mL) was directly collected from the heart in a pre-chilled syringe and transferred to a pre-chilled Eppendorf Protein LoBind tube containing EDTA (40 µL, 50 mM Na_2_EDTA solution) and placed on ice. Samples were processed as previously described [47] and were then analyzed by radioanalytical HPLC. A Symmetry-Shield RP18 (5 µm, 3.9 mm × 20 mm) column (Waters, Eschborn, Germany) was eluted at a 1 mL/min flow rate with the following gradient system (system 2): 100% A/0% B to 60% A/40% B in 40 min; A = 0.1% TFA in H_2_O and B = MeCN.

#### 2.3.2. Biodistribution of [^99m^Tc]Tc-DT11 in PC-3 Tumor-Bearing Mice

The biodistribution of [^99m^Tc]Tc-DT11 was studied in male severe combined immunodeficiency (SCID) mice (11 mice 6-weeks of age on arrival date, 18 ± 4 g, NCSR “Demokritos” Animal House, Athens, Greece) bearing PC-3 xenografts in their flanks. Tumor induction was achieved by subcutaneous injection of a PC-3 cell suspension (12 × 10^6^ cells in 150 µL PBS) in their flanks. The animals were kept under aseptic conditions and, 3 weeks later, developed palpable masses at the inoculation sites (150 ± 50 mg); biodistribution was then conducted [36]. On the day of the experiment, animals were divided into three groups: controls (*n* = 4), Entresto^®^ (*n* = 4) and block (*n* = 3). All mice were injected in the tail vein with a bolus of [^99m^Tc]Tc-DT11 (100 µL, 185 kBq, 5 pmol total peptide in vehicle). In the Entresto^®^ group, animals orally received a pre-prepared dose of the drug (12 mg/200 μL per animal) 30 min in advance (for in situ NEP inhibition), while, in the block group, mice were co-injected with an excess of NT (100 µg; for in vivo NTS_1_R blockade) [47]. All animals were euthanized at 4 h pi and dissected. Blood was collected from the heart, and tumors, organs and tissue samples of interest were excised and weighted, and their radioactivity content was measured together with the proper standards of the injected activity in the gamma counter. The percent of injected activity per gram (%IA/g) was calculated, and the results were provided as mean %IA/g values ± sd for each group. Statistical analyses were performed as described above.

#### 2.3.3. Biodistribution of [^99m^Tc]Tc-DT11+[^99m^Tc]Tc-DB7 in PC-3 Tumor-Bearing Mice

The biodistribution of an equimolar mixture of [^99m^Tc]Tc-DT11 and [^99m^Tc]Tc-DB7 was studied in male SCID mice (20 mice 6-weeks of age on arrival date, 18 ± 4 g, NCSR “Demokritos” Animal House, Athens, Greece) bearing PC-3 xenografts in their flanks. Tumor inoculation was performed as described above. On biodistribution day, the animals were divided into five separate groups of four: (i) controls, (ii) Entresto^®^ (animals orally received a pre-prepared dose of the drug—12 mg/200 μL per animal—30 min in advance for inducing NEP inhibition), (iii) block 1 (co-injected with an excess of NT—100 µg, for in vivo NTS_1_R-blockade—as detailed in “NTS_1_R-Blockade in [^99m^Tc]Tc-DB7+[^99m^Tc]Tc-DT11 Results: Concerns” in the Supplementary Materials), (iv) block 2 (co-injected with an excess of [Tyr^4^]BBN—50 µg, for in vivo GRPR blockade) and (v) block 3 (co-injected with an excess of NT—100 µg and an excess of [Tyr^4^]BBN—50 µg for a double in vivo NTS_1_R and GRPR blockade). All animals received a bolus containing an equimolar mixture of [^99m^Tc]Tc-DT11 and [^99m^Tc]Tc-DB7 in vehicle (100 µL, containing 185 kBq, 5 pmol of each analog). The animals were euthanized at 4 h pi and the experiment was completed as described above; the calculation of the results and there statistical analysis were performed the same way.

#### 2.3.4. SPECT/CT of [^99m^Tc]Tc-DT11+[^99m^Tc]Tc-DB7 in PC-3 Tumor-Bearing Mice

Three more male SCID mice (6-weeks of age on arrival date, 18 ± 4 g, NCSR “Demokritos” Animal House, Athens, Greece) bearing PC-3 xenografts in their flanks, as described above, were used in a SPECT/CT imaging experiment. The animals orally received Entresto^®^ (12 mg/200 μL per mouse) first and, 30 min later, were injected in the tail vein with a mixture of [^99m^Tc]Tc-DT11 and [^99m^Tc]Tc-DB7 (23 MBq, 1.25 nmol of each in 100 µL vehicle) either alone (2 mice) or together with an excess of NT and [Tyr^4^]BBN (300 µg NT and 50 µg [Tyr^4^]BBN for double in vivo NTS_1_R and GRPR blockade—1 mouse). The animals were euthanized 4 h pi and SPECT/CT images were obtained with the aid of the y-CUBE/x-CUBE systems (Molecubes, Ghent, Belgium), as previously reported [47]. SPECT imaging involved a 40 min protocol; a high-analysis CT protocol (50 kVp) followed. For reconstruction of the SPECT images, the MLEM method, with a 250 µm voxel and 100 iterations, was applied, while, for CT images, the ISRA method with a 100 µm voxel was used.

Animal experiments were carried out abiding to European and national regulations by trained and authorized personnel only. The installation was licensed (EL 25 BIO exp021), and the applied protocols were approved by the Department of Agriculture and Veterinary Service of the Prefecture of Athens (stability studies #440448, 1 June 2021; biodistribution/imaging studies #440451, 1 June 2021).

## 3. Results

### 3.1. Ligands and Radioligands

Two previously developed NT and BBN analogs carrying an acyclic tetraamine at their N-terminus and their [^99m^Tc]Tc-labeled versions have been further evaluated in prostate cancer models in the present study. These are the side-chain modified [^99m^Tc]Tc-DT11 [47] and [^99m^Tc]Tc-DB7 [36], depicted in Figure 1.

Labeling of DT11 and DB7 with Tc-99m was previously detailed [36,47]. In short, following a 30 min incubation of [^99m^Tc]TcO_4_^−^-generator eluate with each peptide conjugate in an alkaline aqueous medium in the presence of SnCl_2_ (reductant) and citrate anions (transfer ligand) at room temperature, the successful incorporation of the radiometal in the N_4_ chelator was achieved. Quality control comprising high-performance liquid chromatography (HPLC) and instant thin-layer chromatography (iTLC) methods verified a >98% radioligand formation at typical molar activities of 25–80 MBq/nmol (representative radiochromatograms in Figure S1 of the Supplementary Materials). The total of the radiochemical impurities (unreduced [^99m^Tc]Tc^VII^O_4_^−^, [^99m^Tc]Tc^V^-citrate and reduced hydrolyzed [^99m^Tc]Tc^IV^O_2_ × nH_2_O) was found to be below 2%. Accordingly, [^99m^Tc]Tc-DT11 and [^99m^Tc]Tc-DB7 were used without further purification in all subsequent tests. In addition, their integrity was monitored and confirmed prior to the initiation and after the completion of each biological assay.

### 3.2. In Vitro Assays in PC-3 Cells

The uptake and internalization of [^99m^Tc]Tc-DT11 alone or in an equimolar mixture with [^99m^Tc]Tc-DB7 was directly compared by 1 h incubation at 37 °C in prostate adenocarcinoma PC-3 cells, endogenously co-expressing the human GRPR [53] and NTS_1_R [21,27]. For determining the NTS_1_R-specificity of the [^99m^Tc]Tc-DT11 interaction with the cells, cells were incubated without or in the presence of excess NT, and a 1.4 ± 0.2% NTS_1_R-mediated uptake could be established (percentages of total-added activity per well). This was distributed in an internalized fraction of 1.22 ± 0.26% (≈87% of cell-associated activity) and a membrane-bound fraction of 0.18 ± 0.12% (≈13% of cell-associated activity), consistent with a radiolabeled agonist profile [7,9]. Internalization of [^99m^Tc]Tc-DB7 in PC-3 cells was performed as previously reported [36].

**Figure 2:**
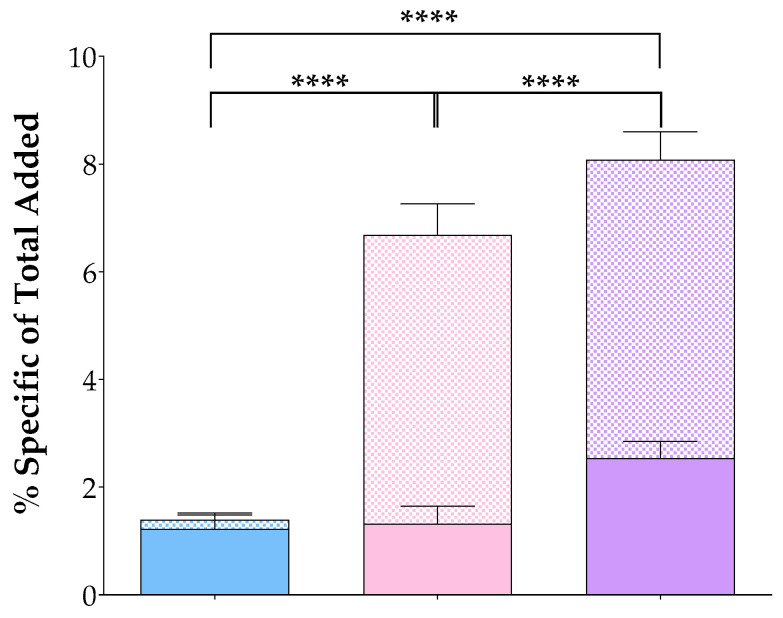
Percentage of receptor-specific radioligand uptake after 1 h incubation at 37 °C in PC-3 cells, comprising internalized (solid bars on the bottom) and membrane-bound fractions (checkered bars on top) corresponding to [^99m^Tc]Tc-DT11 (NTS_1_R-specific: light blue bars on the left), (GRPR-specific: light pink bars in the middle, [36]), and [^99m^Tc]Tc-DT11+[^99m^Tc]Tc-DB7 (NTS_1_R+GRPR-specific: violet bars on the right); results represent mean values ± sd, *n* = 3 (each in triplicate) statistically significant differences are denoted with ****, correspond to *p* < 0.0001.

In the case of the [^99m^Tc]Tc-DT11 and [^99m^Tc]Tc-DB7 equimolar mixture, additional series were used with the mixture incubated in the presence of excess: (i) NT for NTS_1_R-specificity, (ii) [Tyr^4^]BBN for GRPR-specificity, and (iii) an NT and [Tyr^4^]BBN mixture for determining the total NTS_1_R/GRPR-specificity. The obtained values were compared with controls to determine the contribution of each radioligand in cell uptake and internalization. Results on specific values for each analog and their mixture are shown in Figure 2, whereas comprehensive data are summarized in Table 1. The specific uptake of [^99m^Tc]Tc-DT11 in the cells as a part of the radioligand mixture was, again, found to be modest (1.87 ± 0.56% of added activity), not differing from the results obtained when added alone (*p* > 0.05). In comparison, [^99m^Tc]Tc-DB7 showed a much higher cell uptake (7.49 ± 0.46% of added activity; *p* < 0.0001). However, the cell uptake was found to be significantly higher in the radioligand mixture (8.08 ± 0.62% of added activity; *p* < 0.0001 vs. either radioligand uptake). It is interesting to note that the [^99m^Tc]Tc-DB7 behaved as a GRPR antagonist, with the bulk of the specific cell-associated activity remaining bound on the cell membrane (6.40 ± 0.34% membrane-bound fraction vs. 7.49 ± 0.46% sum of membrane-bound + internalized fractions). The contribution of each and both radioligand cell uptake could also be shown by the gradual reduction in total cell-associated activity (10.74 ± 0.61%) by the addition of [Tyr^4^]BBN (single GRPR-blockade–to 4.36 ± 0.51%, *p* < 0.0001;) and the NT+[Tyr^4^]BBN mixture (dual NTS_1_R/GRPR-blockade–further to 2.67 ± 0.21%, *p* < 0.0001), as summarized in Table 1. These results additionally support the contribution of both GRPR and NTS_1_R in the uptake of the [^99m^Tc]Tc-DT11+[^99m^Tc]Tc-DB7 mixture in the cells.

### 3.3. Metabolic Stability

The metabolic stability of [^99m^Tc]Tc-DT11 was previously evaluated in peripheral mice blood at 5 min pi and was found to significantly increase after treatment of mice with the antihypertensive drug Entresto^®^, serving as a source of the potent and specific NEP inhibitor sacubitrilat [47,49,50,51]. The lateral albumin-binding chain at Lys^7^ of [^99m^Tc]Tc-DT11 improved its resistance to the proteolytic action of ACE [47], reported to degrade several NT-based analogs [44,45]. On the other hand, the metabolic stability of [^99m^Tc]Tc-DB7 was previously shown to likewise improve by co-injection of PA [36]. In order to use a single, potent and highly specific NEP inhibitor for both radioligands, we herein compared the effect of the registered drug Entresto^®^ (administered by oral gavage 30 min in advance) with the effect of non-authorized for human use PA (co-injected) on the metabolic stability of [^99m^Tc]Tc-DB7 (further detailed in “Metabolic Studies of [^99m^Tc]Tc-DB7 and [^99m^Tc]Tc-DT11 in Mice: Comments–Results” in the Supplementary Materials). Results are summarized in Table 2, verifying similar [^99m^Tc]Tc-DB7 stabilization effects for PA (94.5 ± 1.1% intact) and Entresto^®^ (93.7 ± 2.2% intact; *p* > 0.05). Both methods led to significant metabolic stability improvements vs. controls (70.6 ± 1.1% intact; *p* < 0.0001; (representative radiochromatograms are included in Figure S2 in the Supplementary Materials).

### 3.4. Biodistribution in PC-3 Tumor-Bearing Mice

#### 3.4.1. Biodistribution of [^99m^Tc]Tc-DT11

**Figure 3:**
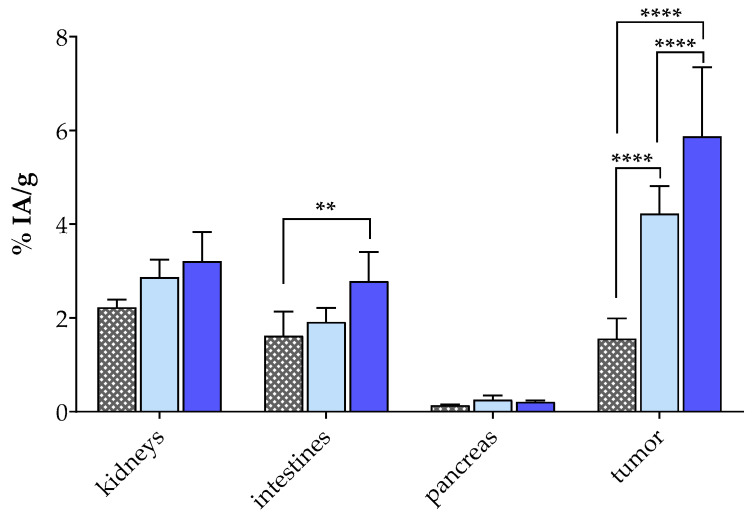
Selected biodistribution data of [^99m^Tc]Tc-DT11 (average %IA/g ± sd at 4 h pi) in PC-3 xenograft-bearing SCID mice; for comparison purposes, bars from left to right in each set correspond to block (animals co-injected excess NT (100 µg), *n* = 3; first bar: gray with square filling), controls (second bar: light blue, *n* = 4) and Entresto^®^ (mice orally receiving Entresto^®^ 30 min before injection of the radioligand, *n* = 4; third bar: dark blue). Values are shown for kidneys, intestines, pancreases and PC-3 tumors; statistically significant differences are denoted: **, for *p* < 0.01 and ****, for *p* < 0.0001.

The biodistribution of [^99m^Tc]Tc-DT11 was first studied in SCID mice bearing PC-3 xenografts at 4 h pi without (controls, *n* = 4), during NEP inhibition induced by oral administration of Entresto^®^ to mice (Entresto^®^, *n* = 4), or during NTS_1_R-blockade by co-injection with excess NT (block, *n* = 3). Cumulative results as average %IA/g ± sd are presented in Table 3, whereas selected values for kidneys, intestines, pancreases and tumors are shown in Figure 3, including statistically significant differences across groups.

The level of background radioactivity (in physiological organs) was favorably low at 4 h after injection of [^99m^Tc]Tc-DT11, in agreement with previous results acquired in another NTS_1_R-positive tumor model in mice [47]. On the other hand, the uptake of [^99m^Tc]Tc-DT11 in the PC-3 xenografts is evident (4.23 ± 0.58%IA/g), surpassing the uptake in all healthy organs and tissues in the animals. This tumor uptake significantly decreased in the group of block mice (to 1.56 ± 0.43%IA/g; *p* < 0.0001), suggesting an NTS_1_R-specific process. During NEP inhibition by Entresto^®^, the uptake in the PC-3 tumors increased compared with controls (to 5.88 ± 1.47%IA/g; *p* < 0.0001), emphasizing the positive effect of metabolically stabilizing the circulating radioligand on tumor targeting [25]. It is interesting to note the absence of considerable increases in the physiological tissues in the Entresto^®^ mice group, favoring the tumor-to-background contrast. The only exception was observed in the intestinal uptake, which was found to be significantly higher in the Entresto^®^ (2.79 ± 0.62%IA/g) than in the block groups of animals (1.62 ± 0.52%IA/g; *p* < 0.01; Figure 3).

#### 3.4.2. Biodistribution of [^99m^Tc]Tc-DT11+[^99m^Tc]Tc-DB7 in PC-3 Tumor-Bearing Mice

**Figure 4:**
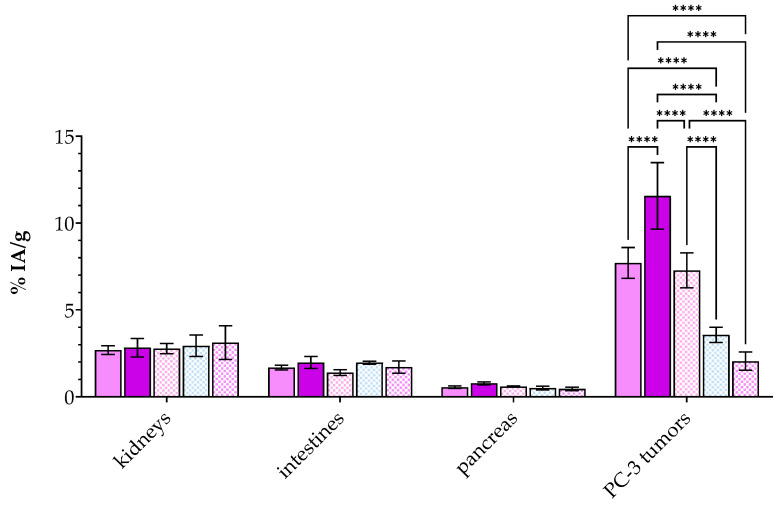
Selected biodistribution data (average %IA/g ± sd; *n* = 4) in SCID mice bearing PC-3 xenografts at 4 h pi of an equimolar [^99m^Tc]Tc-DT11+[^99m^Tc]Tc-DB7 mixture for easy comparison purposes for kidneys, intestines, pancreases and PC-3 tumors. Bars from left to right in each set correspond to controls (first bar: light violet), Entresto^®^ (mice having orally received Entresto^®^ 30 min prior to the injection of the [^99m^Tc]Tc-DT11+[^99m^Tc]Tc-DB7 mixture; second bar: dark violet), block 1 (animals co-injected with 100 µg NT; third bar: pink checkered filling), block 2 (animals co-injected with 50 µg [Tyr^4^]BBN; fourth bar: blue checkered filling) and block 3 (animals co-injected with 100 µg NT and 50 µg [Tyr^4^]BBN; fifth bar: violet checkered filling). Statistically significant differences are denoted: ****, for *p* < 0.0001.

The biodistribution of an equimolar mixture of [^99m^Tc]Tc-DT11 and [^99m^Tc]Tc-DB7 was studied in mice bearing PC-3 xenografts at 4 h pi. To additionally assess the effect of radioligand stabilization on tumor uptake, two separate groups of mice were included, the first comprising non-treated animals (controls) and the second comprising animals who received Entresto^®^ 30 min in advance (Entresto^®^). To assess NTS_1_R-, GRPR- or combined NTS_1_R/GRPR-specificity of uptake, three additional groups of animals were co-injected with NT (block 1), [Tyr^4^]BBN (block 2) or a combination thereof (block 3). It should be noted that, in order to minimize animal distress by co-injection of excess of two potent peptide ligands, the administered amounts were kept low (as detailed in “NTS_1_R-Blockade in [^99m^Tc]Tc-DB7+ [^99m^Tc]Tc-DT11 Results: Concerns” in the Supplementary Materials). Cumulative results as average %IA/g ± sd (*n* = 4) are presented in Table 4, whereas selected values for kidneys, intestines, pancreases and tumors are shown in Figure 4 along with notification of statistically significant differences across groups.

We first observe a favorably low background radioactivity, including the intestines (1.68 ± 0.13%IA/g) co-expressing NTS_1_R and GRPR [54,55] and the GRPR-rich pancreas (0.55 ± 0.08%IA/g) [56]. This pattern is consistent with a rapid background clearance of the radioligand mixture via the kidneys (2.69 ± 0.25%IA/g) and the urinary pathway. On the other hand, the uptake of the [^99m^Tc]Tc-DT11+[^99m^Tc]Tc-DB7 mixture in the implanted PC-3 tumors was clearly superior (7.70 ± 0.89%IA/g) compared with the value achieved after administration of [^99m^Tc]Tc-DT11 alone (Table 3, 4.23 ± 0.58%IA/g; *p* < 0.0001) as well as to the value previously reported for [^99m^Tc]Tc-DB7 in the same model (4.49 ± 1.20%IA/g; *p* < 0.0001) [36]. These findings are in support of the contribution of both [^99m^Tc]Tc-DT11 and [^99m^Tc]Tc-DB7 in the observed uptake in the PC-3 xenografts. It is also interesting to note the tumor-to-kidney ratio increase from ≈1.5 in the case of [^99m^Tc]Tc-DT11 (Table 3) to 2.9 when the [^99m^Tc]Tc-DT11+[^99m^Tc]Tc-DB7 mixture is administered to the animals (Table 4).

Another interesting point is the favorable increase in tumor uptake in the Entresto^®^ group (Table 4, to 11.57 ± 1.92%IA/g; *p* < 0.0001), emphasizing the positive effect of in situ NEP inhibition not only on the stability of both radiopeptides but on their tumor targeting at a later time point as well, as previously reported for a series of NEP-biodegradable peptide radioligands [25]. Again, the tumor values achieved by the [^99m^Tc]Tc-DT11+[^99m^Tc]Tc-DB7 mixture in the Entresto^®^-treated animals was significantly higher than the respective values achieved during NEP inhibition by either [^99m^Tc]Tc-DT11 (5.88 ± 1.47%IA/g in Table 3; *p* < 0.0001) or [^99m^Tc]Tc-DB7 alone (6.10 ± 1.10%IA/g PA-group; *p* < 0.0001–administered in the same dose or molar activity) [36]. At the same time, the uptake in the physiological tissues remained unchanged, resulting in higher contrasts. In the kidneys in particular, a favorable enhancement of the tumor-to-kidney ratio of 4.1 could be reached.

The complementary role of the two NTS_1_R and GRPR tumor-situated receptor targets in the tumor uptake of the [^99m^Tc]Tc-DT11+[^99m^Tc]Tc-DB7 mixture could be additionally confirmed by single (NTS_1_R or GRPR; block 1, 2, respectively; Table 4 and Figure 4) or combined (dual NTS_1_R/GRPR; block 3; Table 4 and Figure 4) in vivo receptor-blockades during NEP inhibition. Thus, the tumor uptake in the Entresto^®^-treated animals was found to be significantly decreased in comparison to block 1 (single NTS_1_R-block; from 11.57 ± 1.92%IA to 7.28 ± 1.00%IA/g; *p* < 0.0001), block 2 (single GRPR-block; from 11.57 ± 1.92%IA to 3.57 ± 0.44%IA/g; *p* < 0.0001) and block 3 (double NTS_1_R/GRPR-block; from 11.57 ± 1.92%IA to 2.05 ± 0.53%IA/g; *p* < 0.0001). It is interesting to observe that the NTS_1_R-blockade with the employed amount of NT (100 µg) was not clearly visible when compared with controls, a finding potentially attributed to the compromised stability of NT (further discussed in “NTS_1_R-Blockade in [^99m^Tc]Tc-DB7+ [^99m^Tc]Tc-DT11 Results: Concerns” in the Supplementary Materials). These findings strongly demonstrate that injection of the [^99m^Tc]Tc-DT11+[^99m^Tc]Tc-DB7 mixture results in a higher tumor uptake compared with individual radioligand injection (Table 3 and Figure 4). They also show that such a favorable effect is mediated by both the NTS_1_R and the GRPR expressed in the prostate cancer lesions in this model.

#### 3.4.3. SPECT/CT of [^99m^Tc]Tc-DT11+[^99m^Tc]Tc-DB7 in PC-3 Tumor-Bearing Mice

**Figure 5:**
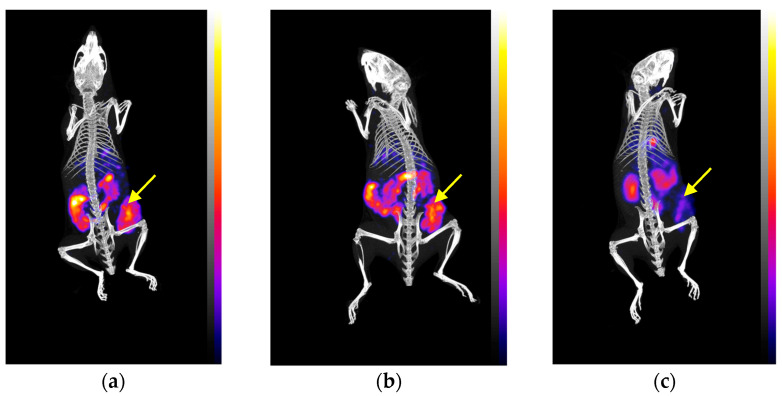
Static whole body SPECT/CT images 4 h pi of an equimolar [^99m^Tc]Tc-DT11+ [^99m^Tc]Tc-DB7 mixture alone (**a**,**b**) or together with 100 µg NT and 50 µg [Tyr^4^]BBN for twin NTS_1_R and GRPR-blockade (**c**). All animals had orally received Entresto^®^ 30 min in advance. Yellow arrows indicate the position of PC-3 xenografts; images correspond to maximum intensity projections, and intensity of uptake is represented by the color scale on the right of each image. Blue corresponds to the lowest and yellow to the highest value.

The visualization of the PC-3 tumors in SCID mice 4 h after injection of a [^99m^Tc]Tc-DT11+[^99m^Tc]Tc-DB7 mixture is shown in Figure 5a,b. The xenografts are clearly delineated along with the kidneys, with some activity being visible in the abdomen. The tumor uptake appears to be significantly reduced in the third mouse (Figure 5c) co-injected with excess NT and [Tyr^4^]BBN for a double NTS_1_R/GRPR-blockade. This qualitative visual comparison is found to be in agreement with a dual-receptor-mediated process demonstrated during biodistribution.

## 4. Discussion

Radiotheranostics have dynamically entered clinical oncology following the successful application of somatostatin analogs in the management of neuroendocrine tumors [2,3,4,5] and are currently expanding to include more clinical indications by exploiting the overexpression of alternative biomolecular targets on different cancer types [1,6,7,8,9]. The concomitant expression of more than one target on cancer lesions [10,11,12,13,14] has also been given due consideration and was first addressed by the introduction of multi-specific radioligands [14,17,18]. This approach has been often linked to sub-optimal pharmacokinetics due to the high molecular weight of peptide multimers, inadvertently leading to increases in background radioactivity levels [22,24]. On the other hand, the application of radiolabeled peptide cocktails has been considered as well; however, to our knowledge, it has not been convincingly addressed yet [18]. Such an approach would require the use of more than one radioligand, typically based on linear peptide sequences which are readily degradable in the biological milieu. That being so, a method to improve their metabolic stability and hence their overall in vivo performance is needed [25].

We decided to investigate the potential advantages–shortcomings of the “radioligand cocktail” approach in human prostate cancer, prompted by the reported co-expression of NTS_1_R and GRPR in this frequently occurring cancer type [16,30,31,33,34]. For such a purpose, we opted for the PC-3 cell line to conduct our cell uptake studies as well as to induce subcutaneous xenografts in mice, as it co-expresses the two targets of interest [27,53]. Based on our previous work on NTS_1_R- and GRPR-directed radioligands, we selected a cocktail consisting of [^99m^Tc]Tc-DT11 (NTS_1_R-specific) [47] and [^99m^Tc]Tc-DB7 (GRPR-specific) [36] to conduct the present preclinical study (Figure 1). Both radioligands are based on linear peptide sequences, modified to easily accommodate the SPECT radionuclide Tc-99m, and were shown to combine a number of characteristics for facilitating direct and reliable comparisons. They both displayed a sub-nanomolar affinity to their respective receptor, a specific uptake in tumor models grown in mice vs. a low radioactivity background and, in addition, improvement in their in vivo performance during in situ inhibition of a single protease, NEP, shown to compromise their metabolic stability [36,47].

Unlike [^99m^Tc]Tc-DB7, [^99m^Tc]Tc-DT11 was not previously tested in prostate cancer models [47]. Hence, we first compared in vitro the uptake of [^99m^Tc]Tc-DT11 in PC-3 cells alone or in an equimolar mixture with [^99m^Tc]Tc-DB7 (Figure 2, Table 1). The results revealed a considerable increase in cell uptake for the radioligand cocktail vs. either radioligand, whereas an alternate single (NTS_1_R or GRPR) or double (NTS_1_R + GRPR) receptor blockade led to partial or maximum loss of uptake in the cells, respectively. These findings illustrate the advantageous contribution of both receptors in enhancing the uptake of radioactivity in the cells. In a similar way, the uptake and overall biodistribution profile of [^99m^Tc]Tc-DT11 was evaluated in PC-3 xenograft-bearing mice, applied alone or in an equimolar mixture with [^99m^Tc]Tc-DB7 (Figure 3 and Figure 4; Table 3 and Table 4, respectively). We likewise observe a significant increase in tumor uptake by going from single to dual radioligand administration (4.23 ± 0.58%IA/g to 7.70 ± 0.89%IA/g at 4 h pi; *p* < 0.0001), whereas the radioactivity background levels remain favorably low (*p* > 0.05). Furthermore, we observe a gradual to maximum decline in the uptake of the [^99m^Tc]Tc-DB7+[^99m^Tc]Tc-DT11 cocktail in the PC-3 tumors in the animals co-injected with double-receptor ligand(s) (NT+[Tyr^4^]BBN for NTS_1_R/GRPR-blockade). These results corroborate the in vitro findings on the synergistic role of NTS_1_R and GRPR on tumor targeting and manifest clear advantages of employing the [^99m^Tc]Tc-DB7+[^99m^Tc]Tc-DT11 cocktail vs. single [^99m^Tc]Tc-DB7 or [^99m^Tc]Tc-DT11 in this prostate cancer model.

In view of the proteolytic action of NEP [36,47] leading to the sub-optimal stability of [^99m^Tc]Tc-DB7 and [^99m^Tc]Tc-DT11 in peripheral mice blood, we decided to explore potential further improvements in the pharmacokinetic profile of the [^99m^Tc]Tc-DB7+[^99m^Tc]Tc-DT11 cocktail when NEP inhibition was induced [25,46]. For such purpose, we selected the potent and selective NEP inhibitor sacubitrilat, which was released into the circulations of the mice after oral administration of the approved antihypertensive drug Entresto^®^ [49,50,52]. Comparison of the Entresto^®^ treatment vs. the previous reported method of co-injecting [^99m^Tc]Tc-DB7 with the NEP inhibitor PA [36,37,39,40] revealed these two methods to be equally effective in stabilizing [^99m^Tc]Tc-DB7. Results of the Entresto^®^ method on the stabilization of [^99m^Tc]Tc-DT11 have been previously reported (Table 2) [47]. The effects of the Entresto^®^ treatment on the PC-3 tumor-bearing mice are included in Figure 3 and Figure 4 and Table 3 and Table 4. Thus, the initial values of [^99m^Tc]Tc-DB7 in the PC-3 tumors significantly increased (4.23 ± 0.58%IA/g to 5.88 ± 1.47%IA/g at 4 h pi; *p* < 0.0001) but were most notably enhanced in the case of the [^99m^Tc]Tc-DB7+[^99m^Tc]Tc-DT11 cocktail (7.70 ± 0.89%IA/g to 11.57 ± 1.92%IA/g at 4 h pi; *p* < 0.0001). At the same time, the radioactivity background levels remained practically unaffected, resulting in a higher tumor-to -background contrast. This finding is well reflected in the favorable increase in tumor-to-kidney ratios from 2.9 (in controls) to 4.1 (in the Entresto^®^ group), highlighting the advantageous application of the [^99m^Tc]Tc-DB7+[^99m^Tc]Tc-DT11 cocktail during in situ NEP inhibition in targeting the prostate cancer lesions. Notably, during NEP inhibition, the specificity of tumor uptake could be clearly illustrated in the significant reduction in the animals treated with single NT (for NTS_1_R-blockade), [Tyr^4^]BBN (for GRPR-blockade) or double-receptor ligand (s) (NT+[Tyr^4^]BBN for NTS_1_R/GRPR-blockade).

The above preclinical results reveal the clear benefits of dual-receptor targeting of cancer by the application of peptide radioligand cocktails. They also emphasize the need for careful selection of individual constituents of such cocktails. Radioligands should have comparable pharmacokinetic properties to ensure maximum tumor-to-background ratios, including rapid clearance, evading the pitfall of inadvertent accumulation in more physiological tissues/organs of the body expressing either one or both receptor targets. It is reasonable to assume that these problems become more severe in the case of higher molecular weight peptide di/multimers instead of cocktails. On the other hand, multimers are likely to be more resistant to degrading peptidases, such as NEP. The stability challenge could be successfully addressed herein in the case of the [^99m^Tc]Tc-DB7+[^99m^Tc]Tc-DT11 cocktail by administration of a suitable protease inhibitor. Nevertheless, the comparison of the [^99m^Tc]Tc-DB7+[^99m^Tc]Tc-DT11 cocktail approach vs. a suitably designed dimer thereof is a task worth pursuing. Yet, all the above issues should be explored beyond prostate cancer in other cancer types, guided by molecular biology and experimental oncology findings, and interesting outcomes should be eventually validated in the clinic. For such prospects, reliable formulations for easy preparation of radioligand cocktails may be additionally required.

## 5. Conclusions

The concomitant expression of peptide receptors in human cancer provides the opportunity for multi-targeting of malignant lesions by employing either multi-specific radioligands or radioligand cocktails for theranostic purposes. In the present work, we explored the advantages and disadvantages of using an equimolar [^99m^Tc]Tc-DB7+[^99m^Tc]Tc-DT11 cocktail for targeting prostate cancer using PC-3 cells and animal models, which are known to co-express the NTS_1_R and the GRPR. The study has clearly confirmed important benefits of using the [^99m^Tc]Tc-DB7+[^99m^Tc]Tc-DT11 cocktail for targeting of the PC-3 implanted tumors in mice, such as a higher tumor uptake and a better tumor-to-background contrast. Further pharmacokinetic profile improvements were evident by applying the [^99m^Tc]Tc-DB7+[^99m^Tc]Tc-DT11 cocktail during NEP inhibition, yielding the most favorable values compared with controls and with either radioligand. These first results need to be complemented by more data from studies on more cancer types and radioligand combinations. They also need to be compared with results from testing multi-specific agents before being eventually validated in the clinic.

## Figures and Tables

**Table 1:** Cell-uptake (internalization + membrane-bound fractions) of the equimolar [^99m^Tc]Tc-DT11+[^99m^Tc]Tc-DB7 mixture during 1 h incubation at 37 °C in PC-3 cells, without or in the presence of excess GRPR or/and NTS_1_R blockers (non-specific), expressed as the average percentage of radioactivity added per well ± sd from three independent experiments performed in triplicate.

		Mixture ^1^	+[Tyr^4^]BBN ^2^	+NT ^3^	+[Tyr^4^]BBN + NT
total/block	membrane-bound	7.04 ± 0.55	1.63 ± 0.13	7.91 ± 0.39	1.50 ± 0.10
	internalized	3.70 ± 0.33	2.73 ± 0.46	2.26 ± 0.13	1.17 ± 0.15
	sum	10.74 ± 0.61	4.36 ± 0.51	10.16 ± 0.45	2.67 ± 0.21
specific	membrane-bound	5.54 ± 0.52 ^4^	0.19 ± 0.15 ^5^	6.40 ± 0.34 ^6^	-
	internalized	2.54 ± 0.32	1.68 ± 0.49	1.09 ± 0.26	-
	sum	8.08 ± 0.62	1.87 ± 0.56	7.49 ± 0.46	-

^1^ Equimolar mixture of [^99m^Tc]Tc-DT11+[^99m^Tc]Tc-DB7; ^2^ radioligand mixture plus excess of [Tyr^4^]BBN added for GRPR-blockade; ^3^ radioligand mixture plus excess of NT added for NTS_1_R-blockade; ^4^ radioligand mixture plus excess of [Tyr^4^]BBN and NT combination added for parallel GRPR and NTS_1_R-blockade; ^5,6^ specific results determined for the [^99m^Tc]Tc-DT11+[^99m^Tc]Tc-DB7 mixture, and the partially blocked groups (either [Tyr^4^]BBN ^5^ or NT ^6^) are those calculated after subtraction of non-specific values during parallel GRPR and NTS_1_R-blockade from the respective total/partially blocked values.

**Table 2:** Results of metabolic stability of [^99m^Tc]Tc-DT11 and [^99m^Tc]Tc-DB7 at 5 min pi in mice circulation without (controls) or 30 min after oral administration of Entresto^®^; for [^99m^Tc]Tc-DB7, additional data corresponding to mice co-injected with PA are included for easy comparison purposes. Results represent the average% intact radioligand ± sd from three different animals per group.

	[^99m^Tc]Tc-DT11 *	[^99m^Tc]Tc-DB7
Control	56.56 ± 5.19%	70.6 ± 1.1% *
Entresto^®^	76.98 ± 3.31%	93.7 ± 2.2%
PA	-	94.5 ± 1.1% *

* Values for [^99m^Tc]Tc-DT11 have been adapted from [47] and for [^99m^Tc]Tc-DB7 control and PA groups from [36].

**Table 3:** Biodistribution of [^99m^Tc]Tc-DT11 in SCID mice bearing PC-3 xenografts at 4 h pi (controls, Entresto^®^-treated and block); data are expressed as average %IA/g values ± sd.

Organs/Tissues	Block (*n* = 3) ^1,2^	Controls (*n* = 4) ^1^	Entresto^®^ (*n* = 4) ^1,3^
Blood	0.52 ± 0.13	1.05 ± 0.30	0.53 ± 0.17
Liver	1.73 ± 0.13	1.39 ± 0.38	1.37 ± 0.24
Heart	0.21 ± 0.03	0.39 ± 0.18	0.17 ± 0.13
Kidneys	0.23 ± 0.17	2.87 ± 0.37	3.21 ± 0.62
Stomach	0.35 ± 0.20	0.38 ± 0.16	0.52 ± 0.20
Intestines	1.62 ± 0.52	1.91 ± 0.30	2.79 ± 0.62
Spleen	0.99 ± 0.30	0.89 ± 0.23	1.27 ± 0.39
Muscle	0.09 ± 0.03	0.16 ± 0.09	0.12 ± 0.03
Lungs	0.76 ± 0.15	1.03 ± 0.42	1.02 ± 1.17
Pancreas	0.14 ± 0.02	0.26 ± 0.09	0.21 ± 0.03
PC-3 Tumor	1.56 ± 0.43	4.23 ± 0.58	5.88 ± 1.47

^1^ Number of animals (*n*) are shown in parenthesis. ^2^ Mice co-injected with excess NT (100 µg) together with the radioligand. ^3^ Mice having received Entresto^®^ (200 μL, 12 mg) per os 30 min in advance.

**Table 4:** Biodistribution of [^99m^Tc]Tc-DT11+[^99m^Tc]Tc-DB7 in SCID mice bearing PC-3 xenografts at 4 h pi (controls, Entresto^®^-treated, and block 1 (for NTS_1_R), 2 (for GRPR) and 3 (for both NTS_1_R and GRPR); data are expressed as average %IA/g values ± sd, *n* = 4.

Organs/Tissues	Controls	Entresto^® 1^	Block 1 ^2^	Block 2 ^3^	Block 3 ^4^
Blood	1.01 ± 0.29	0.72 ± 0.13	1.08 ± 0.13	1.64 ± 0.52	1.41 ± 0.92
Liver	1.27 ± 0.18	1.09 ± 0.15	1.37 ± 0.08	1.81 ± 0.45	1.88 ± 0.44
Heart	0.44 ± 0.11	0.30 ± 0.12	0.39 ± 0.13	0.52 ± 0.19	0.63 ± 0.53
Kidneys	2.69 ± 0.25	2.83 ± 0.53	2.78 ± 0.30	2.93 ± 0.62	3.12 ± 0.97
Stomach	0.58 ± 0.19	0.51 ± 0.22	0.52 ± 0.12	0.67 ± 0.30	0.71 ± 0.13
Intestines	1.68 ± 0.13	1.98 ± 0.35	1.40 ± 0.17	1.96 ± 0.08	1.71 ± 0.36
Spleen	0.67 ± 0.12	0.73 ± 0.10	0.76 ± 0.07	0.98 ± 0.20	0.80 ± 0.17
Muscle	0.14 ± 0.04	0.12 ± 0.02	0.15 ± 0.02	0.22 ± 0.10	0.18 ± 0.11
Lungs	0.80 ± 0.15	0.93 ± 0.17	0.89 ± 0.10	1.33 ± 0.35	1.65 ± 1.02
Pancreas	0.55 ± 0.08	0.77 ± 0.09	0.60 ± 0.03	0.50 ± 0.10	0.45 ± 0.10
PC-3 Tumor	7.70 ± 0.89	11.57 ± 1.92	7.28 ± 1.00	3.57 ± 0.44	2.05 ± 0.53

^1^ Mice pre-treated with Entresto^®^ (200 μL, 12 mg received per os 30 min before) as a sacubitrilat source. ^2^ Mice co-injected with excess NT (100 µg for NTS_1_R blockade). ^3^ Mice co-injected with excess [Tyr^4^]BBN (50 µg for GRPR blockade). ^4^ Mice co-injected with excess NT (100 µg) and [Tyr^4^]BBN (50 µg) for twin NTS_1_R/GRPR in vivo blockade.

## Data Availability

Data are contained within the main article and the Supplementary Materials.

## Supplementary Materials

The following supporting information can be downloaded at https://www.mdpi.com/article/10.3390/pharmaceutics16091223/s1. Figure S1: Radio-HPLC chromatograms of radiolabeled [^99m^Tc]Tc-DB7 and [^99m^Tc]Tc-DT11. Figure S2: Radiometabolite patterns of [^99m^Tc]Tc-DB7 and [^99m^Tc]Tc-DT11 without or during NEP inhibition. Comments on the use of Entresto^®^. Potential sub-optimal NTS_1_R-blocking efficacy concerns when using lower NT doses due to mice welfare considerations.
