# Supplementary material for: Diagnosis of Prostate Cancer with a Neurotensin–Bombesin Radioligand Combination—First Preclinical Results
Source: Pharmaceutics. 2024 Sep 19;16(9):1223. doi: 10.3390/pharmaceutics16091223 (PMC11435135; doi:10.3390/pharmaceutics16091223)
Supplement: Supplementary file 1 [file pharmaceutics-16-01223-s001.zip › pharmaceutics-3154655-supplementary.pdf]

pharmaceutics

MDPI

Article

Supplementary Materials

Diagnosis of Prostate Cancer with a Neurotensin–Bombesin Radioligand Combination—First Preclinical Results

Maria Bibika, Panagiotis Kanellopoulos, Maritina Rouchota, George Loudos, Berthold A. Nock, Eric P. Krenning and Theodosia Maina *

Radioanalytical HPLC Results for $[^{99m}Tc]$Tc-DB7 and $[^{99m}Tc]$Tc-DT11

Representative radiochromatograms for the radiolabeled products $[^{99m}Tc]$Tc-DB7 and $[^{99m}Tc]$Tc-DT11 are presented in Figure S1, confirming previous findings for the formation of a single high-purity radioligand [1,2].

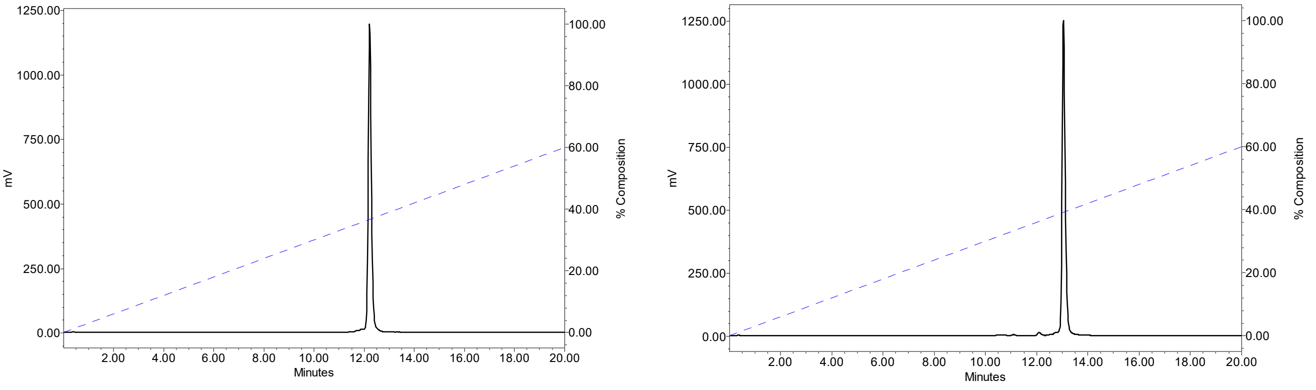

(**a**) (**b**)

Figure S1. Representative radiochromatograms of HPLC analysis of (**a**) $[^{99m}Tc]$Tc-DB7 and (**b**) $[^{99m}Tc]$Tc-DT11 radiolabeled product, verifying the >98% formation of a single radiochemical species applying system 1 (2.1.2. Radiolabeling – Quality Control, in: Materials and Methods).

Metabolic Studies of $[^{99m}Tc]$Tc-DB7 and $[^{99m}Tc]$Tc-DT11 in Mice: Comments – Results

A summary of the metabolic stability results of $[^{99m}Tc]$Tc-DB7 and $[^{99m}Tc]$Tc-DT11 in peripheral mice blood at 5 min pi is summarized in Table 1 of the main article and related radiochromatograms were previously reported [1,2]. Selected radiochromatograms of the HPLC analysis of blood samples collected at 5 min pi of $[^{99m}Tc]$Tc-DB7 and $[^{99m}Tc]$Tc-DT11 are included in Figure S2, showing the in vivo degradation of both radioligands to more hydrophilic species, in agreement to previous reports [1,2]. Thus, coinjection of the potent NEP-inhibitor PA increased the stability of $[^{99m}Tc]$Tc-DB7 from 70.6 ± 1.1% to 94.5 ± 1.1% (representative HPLC results S2 (a) vs. (c)), directly implicating NEP in its degradation to two major radiometabolites [1, 3-5]. On the other hand, although $[^{99m}Tc]$Tc-DT1 mimics were shown to rapidly succumb to the proteolytic action of both NEP and ACE, $[^{99m}Tc]$Tc-DT11 designed for higher in vivo stability, was shown to considerably sustain

degradation by ACE [2, 6-8]. It was shown that in situ inhibition of NEP alone significantly increased the stability of the radiotracer (56.56 ± 5.19% to 76.98 ± 3.31% intact; $p < 0.0001$), whereas the observed further increase of stability by combined NEP and ACE inhibition was not statistically significant (76.98 ± 3.31% to 86.33 ± 1.94% intact; $p > 0.05$) [2]. Thus, [^{99m}Tc]Tc-DT11 was selected as the 2nd and NTS_1R-directed member in the cocktail, namely as sufficiently ACE-resistant [2]. It should be noted that inhibition of additional (vaso)peptidases (e.g. ACE, ECE, aminopeptidases etc.) would eventually complicate clinical translation and should best be avoided, if possible, not only due to increased regulation hurdles. Most importantly, because dual or triple peptidase inhibitors, such as Omapatrilat, present an increased risk for angioedema [9, 10]. Interestingly, the double NEP/ACE-inhibitor Omapatrilat was not-approved by FDA eventually on the basis of angioedema safety concerns [11].

The NEP-inhibition for [^{99m}Tc]Tc-DT11 was achieved by oral gavage of the registered drug Entresto®, reported to in vivo release the highly specific and potent NEP-inhibitor sacubitrilat [2, 12]. The dose of the pill given per mouse was carefully considered taking into account the lack of relevant mice data in published literature. The translation of kinetics from mice to other species, and especially to man, is not linear but much more complex. In addition to inhibition potency, a decisive factor of in situ NEP-inhibition efficacy is bioavailability, namely absorption of the prodrug sacubitril in the gastrointestinal track and delivery into circulation. Such information was eventually acquired from files submitted by Novartis Pharma to regulatory authorities, notably reporting the bioavailability of Entresto® to be strongly species dependent [12-14]. This key information was used to determine an admissible and still effective oral gavage of 12 mg Entresto® pill per mouse (the 24 mg/26 mg sacubitril/valsartan pill weights 200 mg in total and thus, 12 mg of the pill corresponds to 1.44 mg sacubitril) 30 min prior to radioligand injection.

Aiming to employ a single and common NEP-inhibitor for both radioligands of the mixture, we first had to compare the efficacy of the Entresto® vs the PA method in the case of [^{99m}Tc]Tc-DB7, which was found indistinguishable (Table 1 and Figure S2 (c) and (e)).

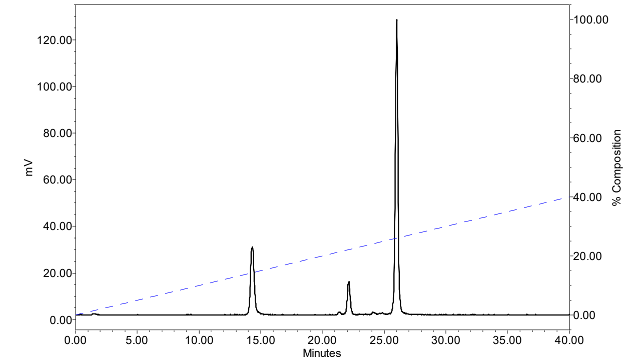

(**a**)

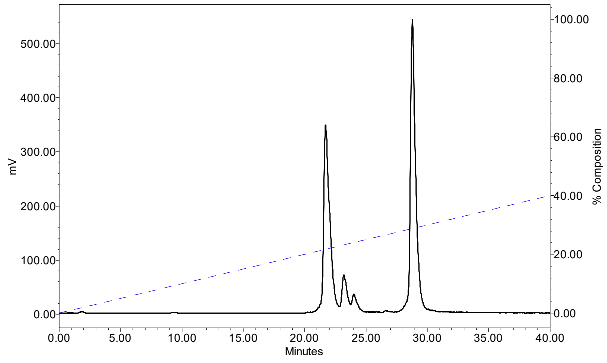

(**b**)

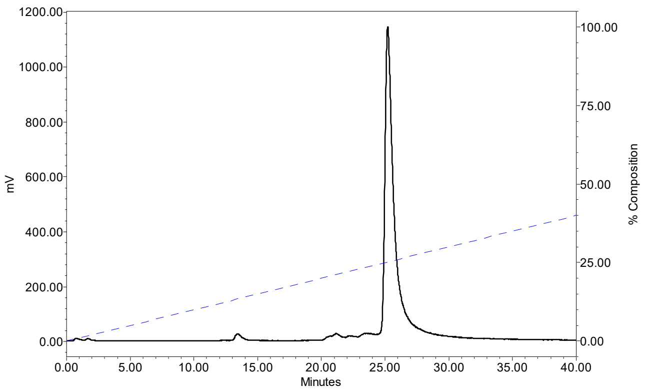

(**c**)

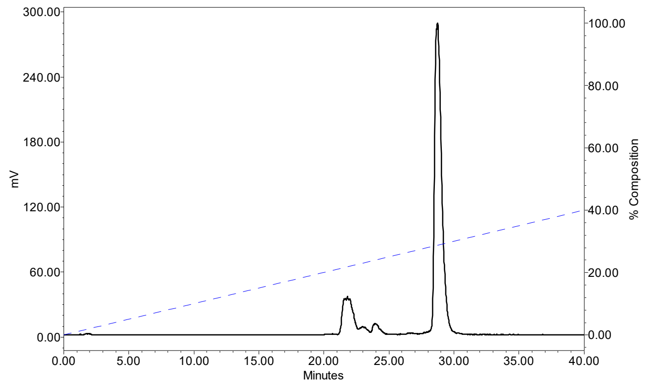

(**d**)

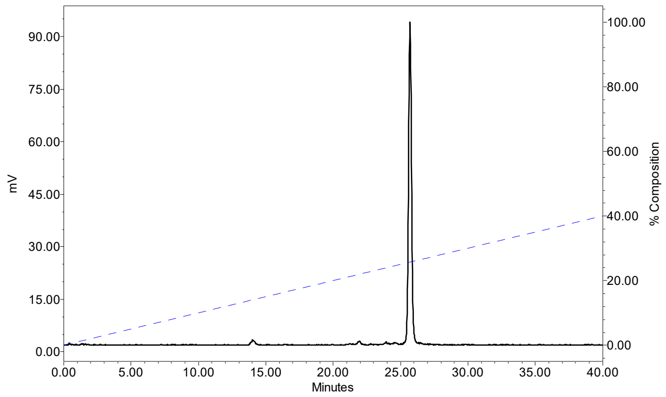

(**e**)

Figure S2. Representative radiochromatograms oh HPLC analysis of blood samples collected 5 min pi in mice of (**a**) [^{99m}Tc]Tc-DB7 and (**b**) [^{99m}Tc]Tc-DT11 (controls), (**c**) [^{99m}Tc]Tc-DB7 co-injected with PA (PA), (**d**) [^{99m}Tc]Tc-DT11 30 min after oral gavage of Entresto® (Entresto®) and (**e**) [^{99m}Tc]Tc-DB7 30 min after oral gavage of Entresto® (Entresto®) applying HPLC system 2 (2. 3. 1. Metabolic Stability in Mice, in: Materials and Methods); chromatograms in (**a**) to (**d**) were adapted from [1,2].

NTS$_1$R-Blockade in [^{99m}Tc]Tc-DB7+ [^{99m}Tc]Tc-DT11 Results: Concerns

In our initial studies with [^{99m}Tc]Tc-DT1 and its mimics for targeting NTS$_1$R-expressing tumors, we were able to effectively block NTS$_1$R in vivo by co-injection of up to 350 µg NT [15]. This amount was necessary, most probably due to the very rapid degradation of NT by NEP, ACE and other peptidases. Alternatively, we were able to achieve NTS$_1$R-blockade with 100 µg NT during ACE/NEP-inhibition, which allowed enough integer NT to reach and block the tumor-situated target [2]. In the first case, we had observed

discomfort in the animals being injected with this high amount of NT. This has posed considerable concerns in the design of the blocking experiment in the present study, whereby two potent peptide agonists ([Tyr[4]]BBN+NT) were to be injected together. Therefore, we decided not to exceed the 100 µg NT + 50 µg [Tyr[4]]BBN total dose, even if this would potentially affect NTS_1R-blockade efficacy in vivo.

Sources:

1. Kanellopoulos, P.; Lymperis, E.; Kaloudi, A.; de Jong, M.; Krenning, E.P.; Nock, B.A.; Maina, T. [^{99m}Tc]Tc-DB1 Mimics with different-length PEG spacers: Preclinical comparison in GRPR-positive models. *Molecules* **2020**, *25*, doi:10.3390/molecules25153418.
2. Kanellopoulos, P.; Nock, B.A.; Rouchota, M.; Loudos, G.; Krenning, E.P.; Maina, T. Side-chain modified [^{99m}Tc]Tc-DT1 mimics: A comparative study in NTS_1R-positive models. *Int. J. Mol. Sci.* **2023**, *24*, doi:10.3390/ijms242115541.
3. Nock, B.A.; Maina, T.; Krenning, E.P.; de Jong, M. "To serve and protect": Enzyme inhibitors as radiopeptide escorts promote tumor targeting. *J. Nucl. Med.* **2014**, *55*, 121-127, doi:10.2967/jnumed.113.129411.
4. Lymperis, E., Kaloudi, A., Sallegger, W., Bakker, I.L.. Krenning, E.P., de Jong, M., *et al*. Radiometal-dependent biological profile of the radiolabeled gastrin-releasing peptide receptor antagonist SB3 in cancer theranostics: Metabolic and biodistribution patterns defined by neprilysin. *Bioconjug. Chem*, **2018**, *29* (5), 1774–84. doi: 10.1021/acs.bioconjchem.8b00225.
5. Kanellopoulos, P.; Kaloudi, A.; Rouchota, M.; Loudos, G.; de Jong, M.; Krenning, E.P.; Nock ,B.A.; Maina, T. One step closer to clinical translation: enhanced tumor targeting of [^{99m}Tc]Tc-DB4 and [^{111}In]In-SG4 in mice treated with Entresto. *Pharmaceutics*. **2020**, *12* (12), 1145. doi: 10.3390/pharmaceutics12121145.
6. Kanellopoulos, P.; Kaloudi, A.; Jong, M.; Krenning, E. P.; Nock, B. A.; Maina, T., Key-protease inhibition regimens promote tumor targeting of neurotensin radioligands. *Pharmaceutics* **2020,** *12* (6). 10.3390/pharmaceutics12060528.
7. Kanellopoulos, P.; Nock, B. A.; Krenning, E. P.; Maina, T., Optimizing the profile of [^{99m}Tc]Tc-NT(7-13) tracers in pancreatic cancer models by means of protease inhibitors. *Int. J. Mol. Sci.* **2020,** *21* (21). 10.3390/ijms21217926.
8. Kanellopoulos, P.; Nock, B.A.; Krenning, E.P.; Maina, T. Toward stability enhancement of NTS_1R-targeted radioligands: Structural interventions on [^{99m}Tc]Tc-DT1. *Pharmaceutics* **2023**, *15*, doi:10.3390/pharmaceutics15082092.
9. Barranco, W. Omapatrilat in: *xPharm: The Comprehensive Pharmacology Reference*, Editor(s): S.J. Enna, David B. Bylund, Elsevier, 8 January 2008, Pages 1-4, doi.org/10.1016/B978-008055232-3.63515-9.
10. Scriabine, A. Chapter 6.32 - Hypertension In: Comprehensive Medicinal Chemistry II, Editor(s): John B. Taylor, David J. Triggle, Elsevier, 2007, Pages 705-728, doi.org/10.1016/B0-08-045044-X/00194-2.
11. Omapatrilat. Available online: https://go.drugbank.com/drugs/DB00886
12. Gu, J.; Noe, A.; Chandra, P.; Al-Fayoumi, S.; Ligueros-Saylan, M.; Sarangapani, R.; Maahs, S.; Ksander, G.; Rigel, D.F.; Jeng, A.Y.; et al. Pharmacokinetics and pharmacodynamics of LCZ696, a novel dual-acting angiotensin receptor-neprilysin inhibitor (ARNi). *J. Clin. Pharmacol.* **2010**, *50*, 401-414, doi:10.1177/0091270009343932.
13. Australian Public Assessment Report for sacubitril / valsartan salt complex, September 2016, Available online: https://www.tga.gov.au/sites/default/files/161013_auspar-sacubitril-valsartan-salt-complex-160923.pdf
14. Center for drug evaluation and research, *Application number:* 207620orig1s000, Pharmacology Review(s): sacubitril/valsartan, Novartis, 29 October 2014, Available online: https://www.accessdata.fda.gov/drugsatfda_docs/nda/2015/207620Orig1s000PharmR.pdf.
15. Maina, T.; Nikolopoulou, A.; Stathopoulou, E.; Galanis, A. S.; Cordopatis, P.; Nock, B. A., [^{99m}Tc]Demotensin 5 and 6 in the NTS1-R-targeted imaging of tumours: Synthesis and preclinical results. *Eur. J. Nucl. Med. Mol. Imaging* **2007,** *34* (11), 1804-14. 10.1007/s00259-007-0489-z.
